# Effects of Host, Sample, and *in vitro* Culture on Genomic Diversity of Pathogenic Mycobacteria

**DOI:** 10.3389/fgene.2019.00477

**Published:** 2019-06-04

**Authors:** Abigail C. Shockey, Jesse Dabney, Caitlin S. Pepperell

**Affiliations:** ^1^Department of Medical Microbiology and Immunology, School of Medicine and Public Health, University of Wisconsin-Madison, Madison, WI, United States; ^2^Department of Physiology, Anatomy and Genetics, University of Oxford, Oxford, United Kingdom; ^3^Department of Medicine, Division of Infectious Diseases, School of Medicine and Public Health, University of Wisconsin-Madison, Madison, WI, United States

**Keywords:** *Mycobacterium tuberculosis*, *Mycobacterium bovis*, genome evolution, within-host adaptation, bacterial genomics, hybridization capture

## Abstract

*Mycobacterium tuberculosis* (*M. tb*), an obligate human pathogen and the etiological agent of tuberculosis (TB), remains a major threat to global public health. Comparative genomics has been invaluable for monitoring the emergence and spread of TB and for gaining insight into adaptation of *M. tb*. Most genomic studies of *M. tb* are based on single bacterial isolates that have been cultured for several weeks *in vitro*. However, in its natural human host, *M. tb* comprises complex, in some cases massive bacterial populations that diversify over the course of infection and cannot be wholly represented by a single genome. Recently, enrichment via hybridization capture has been used as a rapid diagnostic tool for TB, circumventing culturing protocols and enabling the recovery of *M. tb* genomes directly from sputum. This method has further applicability to the study of *M. tb* adaptation, as it enables a higher resolution and more direct analysis of *M. tb* genetic diversity within hosts with TB. Here we analyzed genomic material from *M. tb* and *Mycobacterium bovis* populations captured directly from sputum and from cultured samples using metagenomic and Pool-Seq approaches. We identified effects of sampling, patient, and sample type on bacterial genetic diversity. Bacterial genetic diversity was more variable and on average higher in sputum than in culture samples, suggesting that manipulation in the laboratory reshapes the bacterial population. Using outlier analyses, we identified candidate bacterial genetic loci mediating adaptation to these distinct environments. The study of *M. tb* in its natural human host is a powerful tool for illuminating host pathogen interactions and understanding the bacterial genetic underpinnings of virulence.

## Introduction

Tuberculosis (TB) is the leading cause of death worldwide due to an infectious disease (World Health Organization [WHO], 2018). Among the tools brought to bear to understand and tackle the TB pandemic, comparative genomics has received increased attention following the development of affordable, high throughput sequencing technologies. For example, comparative genomic methods have been used to investigate the spread of TB at regional (e.g., Martin et al., 2018) and global (e.g., O’Neill et al., in press) scales and to identify drug resistance loci (e.g., Farhat et al., 2013; Zhang et al., 2013; Mortimer et al., 2018).

As a first step to performing analyses of *M. tb* whole genome sequence data, isolation of bacterial DNA typically begins with decongestion of putatively infected sputum and transfer to artificial media. Sputum samples that harbor *M. tb* are then enriched for *M. tb* through growth in axenic culture for multiple weeks before DNA extraction can be performed (Fadzilah et al., 2009). Although routine, the microevolutionary dynamics of this process are not well characterized.

Recent methodological advances have enabled enrichment of target DNA molecules from within complex backgrounds. DNA enrichment via hybridization capture has become a standard procedure for recovering genomic regions of interest from genetically homogenous mixtures, and even full genomes from complex metagenomic backgrounds (Christiansen et al., 2014; Enk et al., 2014; Clark et al., 2018). Indeed, enrichment via hybridization capture has recently been investigated as a rapid TB diagnostic, circumventing lengthy culturing procedures and enabling the recovery of *M. tb* genomes directly from infected sputum (Brown et al., 2015; Doyle et al., 2018).

In addition to TB diagnostics, the ability to recover genomes directly from infected tissues has important implications for the field of *M. tb* comparative genomics. Most studies have relied on 1:1 comparisons of representative genomes from single bacterial strains isolated via axenic culture. However, *M. tb* populations within hosts are composed of potentially billions of bacterial cells that diversify over the course of infection and cannot be wholly represented by a single genome (O’Neill et al., 2015; Trauner et al., 2017). Capturing bacterial genomic material directly from sputum enables a more direct analysis of *M. tb* genetic diversity during infection.

Here we used metagenomic and Pool-Seq approaches to compare genome-wide sequence data from *M. tb* and *M. bovis* populations isolated from paired sputum and culture samples. Our results suggest genetic diversity is reshaped during *in vitro* culture of bacterial populations and we propose candidate loci mediating differential adaptation to these distinct environments.

## Materials and Methods

### Sample Collection

We analyzed whole genome sequence data from a previously published study in which *M. tb* DNA was captured directly from infected sputum samples (accession code PRJEB9206) (Brown et al., 2015). TB treatment data were not provided for samples included in Brown et al.; according to the text, at least some of the patients had received TB treatment prior to sample collection. We obtained an additional five residual sputum samples from the Wisconsin State Laboratory of Hygiene. Four of these samples were taken from a single TB patient over a 48-h period. These were the first samples collected from this patient. The presence of *Mycobacterium bovis* (*M. bovis*) in these four had been confirmed through positive MGIT cultures. The fifth sputum sample was a pool taken from numerous TB negative patients.

### Sample Preparation and Sequencing

Two 500 ul aliquots were taken from each positive sputum sample. One aliquot was used directly for DNA extraction, while the second aliquot was used for inoculation into 10 ml of Middlebrook 7H11 broth in T-25 culture flasks. Cultures were incubated at 37°C for 3 weeks or longer until growth was visible.

DNA extractions from all samples, including a 500 ul aliquot from the negative sputum sample, were performed following the protocol described in Brown et al., with some modifications. Briefly, the 500 ul aliquots of decongested sputum, and 5 ml of culture were spun down for 5 min at maximum speed on a benchtop centrifuge to pellet cells. The supernatant was discarded, and the sediment then resuspended in 300 ul TE buffer and transferred to 2 ml tubes with 250 ug of 0.1 mm glass beads. Samples were incubated at 80°C for 50 min, and then frozen at -80°C overnight. After thawing, tubes were vortexed for 3 min and spun down, followed by the addition of 10 ul Mutanolysin and 1-h incubation at 37°C. Following incubation, samples were centrifuged at max speed on a benchtop centrifuge and purified with the DNeasy Blood and Tissue kit (Qiagen) and eluted in 100 ul volumes. All extractions were performed in a BSL-3 laboratory.

For the sputum samples, 50 ul of each extract was sheared using the Covaris M220 on 250 bp setting. 10 ul of sheared DNA was then used as input for sequencing library preparation using the NEBNext Ultra II kit (New England Biolabs) following manufacturer’s instructions. 1:100 dilutions of each library were quantified via qPCR using Maxima master mix (ThermoFisher). Double indexes (NEB) were then added using AccuPrime Pfx polymerase (ThermoFisher) and the following PCR heating profile: 2 min at 95°C, 15 cycles of 20 s at 95°C, 30 s at 65°C and 1 min 20 s at 68°C, followed by 5 min at 68°C. PCR reactions were purified using MinElute spin columns (Qiagen). 1 ul of each resulting indexed library was then run through one cycle of PCR to remove heteroduplices and purified with MinElute columns. Samples were pooled in equal volumes and negative controls in 1:10 volumes. This pool was quantified on a BioAnalyzer using a DNA 1000 chip and sequenced on 1 lane of a 2 × 125 bp run on a HiSeq 2500.

The culture samples were prepared according the TruSeq Nano DNA LT Library Prep Kit (Illumina Inc., San Diego, CA, United States) with minor modifications. Samples were sheared using a Covaris M220 Ultrasonicator (Covaris Inc., Woburn, MA, United States), and were size selected for an average insert size of 550 bp using SPRI bead-based size exclusion. Quality and quantity of the finished libraries were assessed using an Agilent DNA1000 chip and Qubit ^®^dsDNA HS Assay Kit, respectively. Libraries were standardized to 2 nM.

### Sequence Processing

We processed fastq files for all samples using our reference guided assembly pipeline^1^. Briefly, adapters and low-quality bases were trimmed using Trim Galore!^2^ and aligned to either the *M. tb* H37Rv (Brown et al., 2015 samples and the negative sputum sample) or *M. bovis* AF2122 (samples from the Wisconsin State Laboratory of Hygiene) reference genomes using BWA mem (Martin, 2011; Li, 2013). SAM files were converted to bam format and sorted using Samtools followed by duplicate removal with Picard^3^ and local realignment with GATK (Li et al., 2009; DePristo et al., 2011).

Aligned sequences were taxonomically classified using Kraken and the RefSeq bacterial, viral and archaea databases as implemented in Kraken’s standard database build (Wood and Salzberg, 2014; O’Leary et al., 2016). Paired-end sequences where one or both sequences were not assigned to the *Mycobacterium* genus or lower were removed from the aligned sequences using Picard. Indels, repetitive regions (including PE/PPE genes), mobile elements, as well as rRNA and tRNA genes were removed from samples using PoPoolation2 (Schlötterer et al., 2011; Supplementary Table S1). We identified and removed indels present in each sample using PoPoolation (Kofler et al., 2011). We used VCF files generated with Samtools to identify strand-bias positions in each sample, which were removed across all samples.

### Estimates of Nucleotide Diversity

We estimated genetic diversity for each sample independently using the Pool-seq approach implemented in PoPoolation2. Following O’Neill et al. (2015), we randomly subsampled (*n* = 10) read data from each sample to a uniform 50× coverage to limit the effects of differential coverage across samples. Using these subsampled data with uniform coverage, we then calculated nucleotide diversity (π), Watterson’s theta (θ_w_) and Tajima’s D in 100 kb sliding windows across the genome in 10 kb steps (O’Neill et al., 2015). Additionally, we calculated π and θ_w_ for each gene using gene annotations based on the *M. tb* H37Rv and *M. bovis* AF2122 reference genomes. Following recommendations and rationale described in O’Neill et al., we required at least 50% coverage of each region and a minimum allele count of 2. Pool-size was set at 10,000. We calculated the genome-wide averages of nucleotide diversity in sputum and culture samples for each patient as the mean of the sliding windows of diversity (100 kb windows, 10 kb steps). We performed a paired *t*-test on these genome-wide values of nucleotide diversity in sputum and culture for each patient.

### Identification of Windows of Overlap in Nucleotide Diversity

We identified regional peaks in nucleotide diversity across the genome. Using nucleotide diversity from the sliding-window analysis, we calculated a *z*-score and *p*-value for each window in sputum and culture for each patient. We performed FDR correction, setting a *p*-value cutoff of 0.05. Windows were defined as overlapping if they were present in > 1 patient. Code available on https://github.com/AbigailShockey/sputum.

### Identification of Outlier Genes

We identified genes with significant changes in diversity from sputum to culture in each patient using three different approaches. Method 1: we performed linear regression of nucleotide diversity (π) per gene in sputum versus culture for each patient (Yahara et al., 2016). We calculated Cook’s distance (*D*_i_) from the regression line for each gene and used a threshold of > 4 times the mean of *D*_i_ to define outlier genes in each patient. Method 2: for each patient and each gene we calculated the fold-change in nucleotide diversity between sputum and culture samples (i.e., nucleotide diversity in sputum/nucleotide diversity in culture). We performed z-transformation of these values and calculated a *p*-value for each gene. For genes with non-zero diversity in sputum and zero diversity in culture, we calculated a *z*-score and *p*-value for the difference in nucleotide diversity between these sample types. We used FDR correction for multiple testing, setting a cutoff of 0.05 to identify outliers. Method 3: treating sputum and culture pairs as two different populations, we calculated *F*_ST_ per gene using PoPoolation2. We required a minimum allele count of 3, minimum coverage of 10 and maximum coverage of 350. Pool-size was set at 10,000. Genes that were masked (either insufficient coverage or within the bounds of removed regions described in Supplementary Table S1) in the gene-wise estimates of nucleotide diversity described above were excluded from these analyses. We performed a Fisher’s exact test with FDR correction to assess significance for *F*_ST_ values from each gene, setting a *p*-value cutoff of 0.01. Code available on https://github.com/AbigailShockey/sputum.

### Lineage Typing

We used SNP-IT (Lipworth et al., 2019) to perform lineage typing for our sample of *M. tb*. Briefly, we used bcftools to call consensus sequences from our sputum and culture samples of *M. tb*. We required a minimum read and mapping quality of 20. We masked indels, repetitive regions (including PE/PPE genes), mobile elements, as well as rRNA and tRNA genes in the consensus sequences (Supplementary Table S1) using scripts found at https://github.com/tatumdmortimer/formatConverters/blob/master/maskFasta.py. We performed lineage typing on the masked consensus sequences.

### Identification of Mixed Infections

In order to investigate the possibility of mixed infection, we looked for overlap between sites defined as variable in our analyses and lineage-defining positions from (Coll et al., 2014). Of the 6,915 positions proposed by Coll et al., 47 were masked in our analyses due to not meeting quality control thresholds. We did not observe consistent patterns of variation at the remaining 6,868 positions to suggest that the samples derived from infections that contained mixtures of lineages (Supplementary Table S2).

### Data Availability

The *M. tuberculosis* sequence data from Brown et al. (2015) are publicly available in the Sequence Read Archive under BioProject Accession Code PRJEB9206. The *M. bovis* sequence data are available under BioProject Accession Code PRJNA532927.

### Ethics Statement

Newly sequenced data in this study were obtained from residual clinical samples at the State Lab of Hygiene. We did not collect any data or samples for research purposes nor was routine clinical care altered by this study. This study was reviewed and approved by the UW-Madison Health Sciences Institutional Review Board.

## Results

### Removal of Putative Contaminating Sequences With Metagenomic Filtering

To address the possibility of background contamination with high sequence similarity to the *M. tb* H37Rv or *M. bovis* AF2122 reference genomes, we performed metagenomic filtering on aligned reads from all samples. We used Kraken (Wood and Salzberg, 2014) to assign each read to a taxon and removed reads not assigned to the *Mycobacterium* genus or a species within it.

**FIGURE 1:**
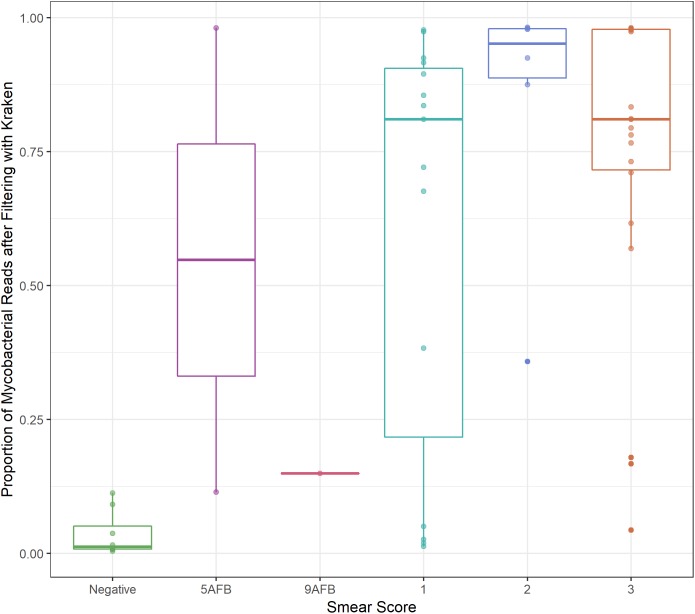
Smear score. Boxplot of proportion of sequencing data retained after filtering (*y*-axis) versus sputum smear score (*x*-axis) for *M. tb* sputum samples from Brown et al. Thresholds for smear score of potentially infected sputum are 0 AFB/100 fields: smear negative, 1–9 AFB/100 fields: actual number of AFB seen on slide, 10–99 AFB/100 fields: 1+, 1–10 AFB/field in 50 fields: 2+,>10 AFB/field in 20 fields: 3+, where AFB corresponds to the number of acid-fast bacilli present.

Between 8–99% of aligned sequences were removed from the sputum samples, with 9 of the 35 samples losing more than 50% of aligned sequences (Supplementary Table S3). The *M. tb* sputum samples from Brown et al. (2015) were published with associated smear scores ranging from negative to 3+. The percent of sequences remaining after filtering increased with smear score (Figure 1). This increase was significant for samples with a smear score ≥ 1+ when compared to samples with a negative sputum score (ANOVA, *p*-value < 0.01; Supplementary Table S4) suggesting that some of the variation in the number of sequences removed can be attributed to the severity of infection. However, the variation in the *M. bovis* sputum samples taken over a 48-h period (1–31% sequences retained after filtering) indicate the degree of stochasticity when sampling repeatedly from a single patient (Supplementary Table S3).

We applied this filter to culture samples, which allowed us to assess the stringency of this step, as these samples should have minimal contaminating sequences. In samples with >50× starting coverage, less than 3% of aligned sequences were removed by the filter. The filtered sequences likely arise from low levels of contamination or sequences in conserved regions that can’t be confidently assigned (Supplementary Table S3).

Similarly, we applied the filter to a pool of negative sputum from patients without TB. Since there should be no sequences belonging to *Mycobacterium* genus in this sample, any sequences carried through must come from background contamination not detectable by alignment or the metagenomic filter. Only 0.3% of starting sequences could be aligned to either the *M. tb* or *M. bovis* reference genome. From those, 98% were removed in the metagenomic filter step, indicating that the filter, together with alignment, is efficient at removing potential contaminating sequences contributed by metagenomic background (Supplementary Table S3).

In conjunction with the metagenomic filter, we also removed indels, repetitive regions (including PE/PPE genes), rRNA and tRNA genes, and mobile elements (Supplementary Table S1). Together these filters lead to an average reduction in genome wide coverage of 14% in culture samples, and 34% in sputum samples (Supplementary Table S2). Only samples with 50× or greater final coverage were included in subsequent analyses.

### Effect of Sampling and Sample Type on Bacterial Genetic Diversity

**FIGURE 2:**
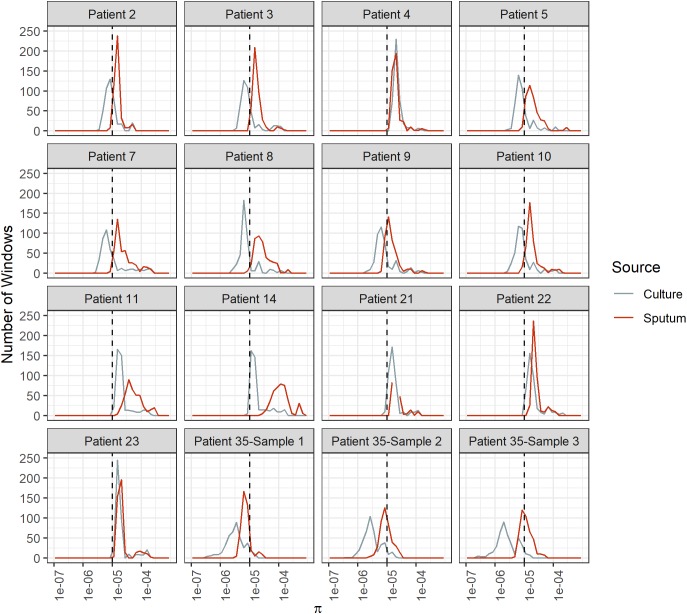
Bacterial diversity across genome windows in sputum and culture samples. Frequency (*y*-axis) of nucleotide diversity (π, *x*-axis) in sliding windows across the genome (windows = 100 Kb, step-size = 10 Kb). Diversity varies among patients as well as among samples from the same patient, as shown by the differences in the shapes of these distributions. Sputum samples exhibit more variability among patients, and diversity is generally higher than it is for culture samples. Sputum and culture in red and gray, respectively. Dotted line at π = 1e-5.

**FIGURE 3:**
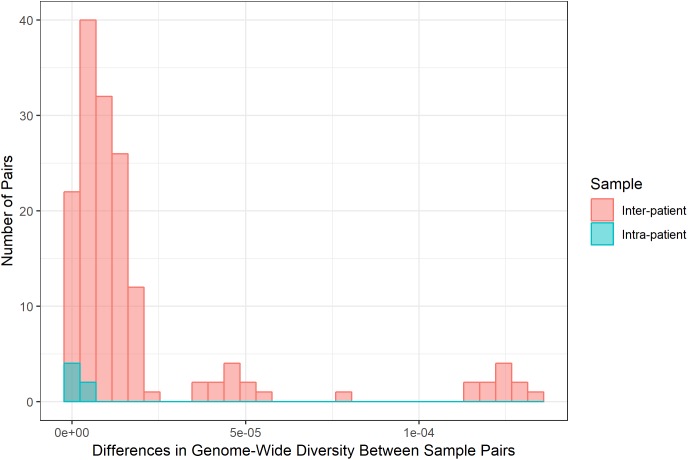
Differences in bacterial diversity among inter-patient and intra-patient pairs of samples. Frequency (*y*-axis) of absolute difference in genome-wide nucleotide diversity (average π across sliding windows, *x*-axis) between samples. We calculated the absolute difference in genome-wide π for all possible pairs of sputum samples and all possible pairs of culture samples for inter- and intra-patient samples (sputum versus sputum and culture versus culture). Inter- and intra-patient differences pictured in coral and teal, respectively. Differences among samples from the same patient are similar to the bulk of comparisons between patients. Some between-patient pairs exhibit extreme differences in diversity.

Diversity of *M. tb* samples varies among patients (Figure 2). We did not find any evidence to suggest this was driven by bacterial lineage (Supplementary Table S3). Nucleotide diversity is more variable among sputum samples, where genome-wide values span an order of magnitude, whereas culture samples are more homogenous. The distribution of pairwise differences among samples from the same patient is nested within the distribution for differences between patients (Figure 3). This is consistent with a substantial impact of sample to sample variation on bacterial genetic diversity, similar to the observed effect of sampling on the amount of target sequencing data recovered (Supplementary Table S3). The distribution of windows of nucleotide diversity (π) across the genome varied across comparisons from the same patient, further reflecting the effects of sampling (ANOVA *p*-value < 0.05 for sample 1–3 and sample 2–3 comparison; NS for comparison of sample 1 and 2).

**FIGURE 4:**
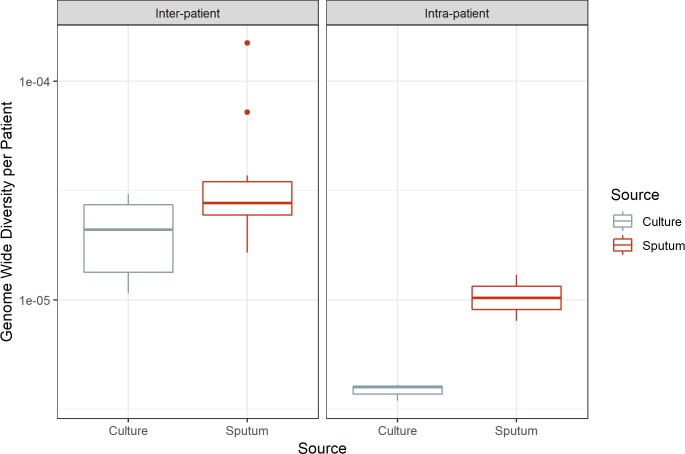
Genome-wide diversity in sputum and culture. Average genome-wide nucleotide diversity (π) in sputum and culture samples. Sputum and culture pictured in red and gray, respectively. Left box, inter-patient samples; right box, intra-patient samples. Medians 2.77e-05 and 2.10e-05, 1.02e-05 and 3.98e-06 for sputum and culture in inter-patient and intra-patient samples, respectively.

Despite inter- and intra-patient variability, there is a consistent pattern of greater diversity in sputum versus culture: genome-wide π is significantly greater in sputum (paired *t*-test, *p*-value = 0.029, 0.028 for inter-patient and intra-patient samples, respectively; Figure 4). This is indicative of a systematic loss of diversity during growth in culture.

Values of θw are generally higher than π in all samples, indicating an abundance of low frequency variants (Supplementary Figures S1, S2). Tajima’s D is uniformly low across the genome (Supplementary Figure S3). Average Tajiima’s D values are lower in sputum samples for all but one patient.

It is possible that background contamination not removed during alignment or metagenomics filtering contributed to observed differences in diversity between sputum and culture. Homologous sequences from non-mycobacteria present in the lungs or respiratory tract would not be present in culture and could artificially inflate nucleotide diversity in sputum samples. To address this problem, we sequenced a pool of sputum from multiple patients not displaying symptoms of TB and processed these sequences identically to infected samples. Although 99.9% of sequences were removed, approximately 3,000 sequences passed through our filters. We merged these sequences with those from Patient 14’s culture sample and calculated nucleotide diversity (π) in sliding-windows as described above. Average π in this composite sample was slightly higher than the culture sample alone, but less than the paired sputum sample (π = 2.69e-05, 6.74e-05, and 1.47e-04 in culture, composite, and sputum, respectively). Increases in nucleotide diversity in the composite sample did not mirror the topology of Patient 14’s sputum sample (Supplementary Figure S4). These findings indicate minimal background contamination passes our filters, and this contamination does not drive the patterns of nucleotide diversity seen in sputum samples.

### Regional Patterns of Diversity

To identify regional peaks in diversity across the genome, we calculated a *z*-score and *p*-value for π per window in sputum and culture for each patient. We identified 35 regions of overlapping high π (i.e., present in > 1 patient) between culture samples from different patients, and 34 windows of overlap among sputum samples. There are 17 windows found in multiple patients in both sputum and culture. These windows correspond to two genomic regions (Supplementary Table S5). From the intra-patient samples, there were five windows of overlap in sputum and 12 in culture. No windows were shared between culture and sputum samples from the same patient.

To assess whether changes in diversity between sputum and culture samples occur in specific genomic regions, we calculated the fold-change across the genome as the ratio of π in sputum to π in culture in sliding windows across the genome. For four patients, the diversity of culture and sputum samples was similar. Patterns of diversity in the other patients did not reveal any obvious “hotspot regions” across patients or samples in which culture and sputum exhibited consistent differences (Supplementary Figure S5).

### Patterns of Variation at the Individual Gene Level

**FIGURE 5:**
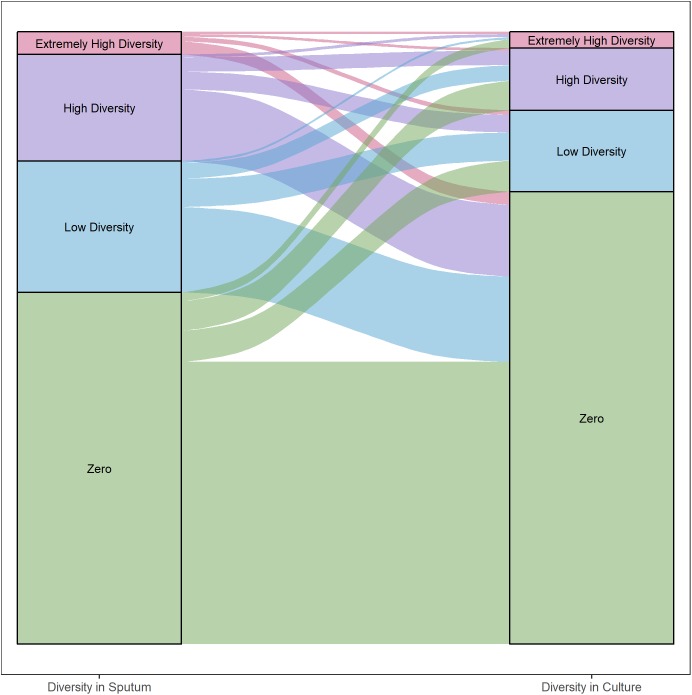
Differences in gene diversity of sputum and culture samples. Alluvial plot of changes in nucleotide diversity (π) per gene in sputum and culture samples (left and right, respectively). Inter- and intra-patient samples were all included. Categories defined by diversity quartiles: Extremely high (≥Q3 + 1.5 × IQR), high (≥Q2 and <Q3 + 1.5 × IQR), low (≥Q1 – 1.5 × IQR and <Q2) and zero diversity. Strata colored by each category. Strata width corresponds to the total number of genes in each category.

We categorized each gene based on differences in nucleotide diversity between sputum and culture. The majority of genes in each patient maintained zero diversity or decreased in diversity (Figure 5, 54 and 32% of total gene content, respectively). Among genes that decreased in diversity, the majority lost all diversity in culture. As with the findings described above, these results suggest a significant loss in bacterial diversity occurs following growth in culture.

**FIGURE 6:**
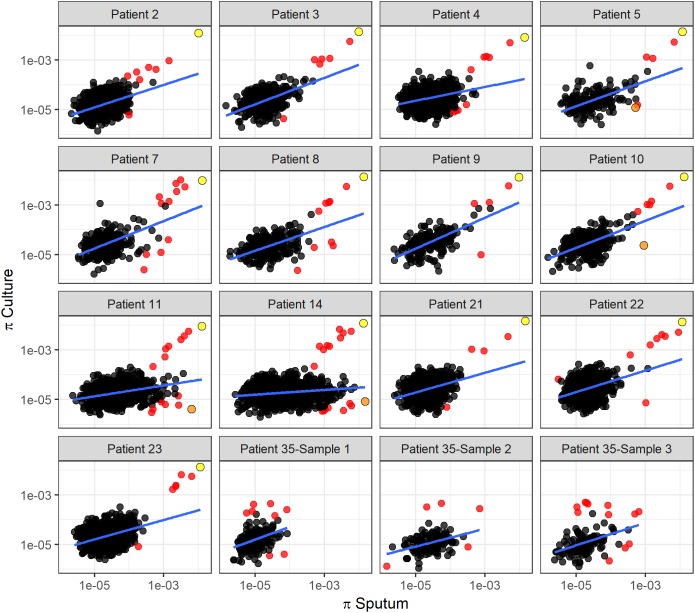
Linear regression of gene diversity in culture versus sputum. Nucleotide diversity (π) per gene in sputum (*x*-axis) versus culture (*y*-axis) for each patient. Each dot corresponds to a gene identified in sputum and culture samples. Regression line is shown in blue. Red dots mark outliers as identified with Cook’s distance from the regression line (i.e., genes with Cook’s distance > 4 times the mean of observed distances). We used the *F*-test of overall significance to assess the fit of a linear regression model to the observed data; no *F*-test had a *p*-value > 0.05. The slope of the regression line varies substantially among patients. Two genes identified as outliers in this linear regression analysis are specifically highlighted: Rv2020c, marked in yellow, appeared as an outlier in all 13 TB patients. *nrdE*, marked in orange, also appeared repeatedly as an outlier but in a different part of the distribution.

To identify specific genes with marked changes in diversity between sputum and culture, we performed linear regression of gene diversity in the two sample types, for each patient and sample. We identified 49 outlier genes in *M. tb*, 17 of which were found across more than one patient (Figure 6, Table 1, and Supplementary Table S6). Remarkably, Rv2020c was an outlier in all 13 patients.

**Table 1:** *M. tb* genes with extreme patterns of variation across multiple patients and multiple measures.

Gene	lm	*z*-score	Z0	*F*_ST_
Rv2020c (hypothetical)	13	0	0	6
Rv1318c	11	0	0	11
Rv1319c	9	0	0	6
Rv3109 (*moaA1*)	11	0	0	8
Rv 1267c (*embR*)	10	0	0	5
Rv2351c (*plcA*)	5	0	0	5
Rv2350c (*plcB*)	5	0	0	4
Rv2082 (hypothetical)	3	0	0	3
Rv2081c (conserved transmembrane protein)	2	0	0	2
Rv3051c (*nrdE*)	4	6	2	7
Rv0338c	2	1	0	2
Rv0684 (*fusA1*)	2	1	2	3
Rv1164 (*narI*)	2	1	2	5
Rv1327c (*glgE*)	2	1	0	2
Rv1630 (*rpsA*)	2	2	1	2
Rv0667 (*rpoB*)	2	3	1	4
Rv0668 (*rpoC*)	1	1	2	4


*The number of patients in whom each gene was identified as an outlier is shown for linear regression of diversity in sputum versus culture (lm), fold change in diversity (*z*-score) or absolute difference (Z0) and *F*_*ST*_ analyses.*

**FIGURE 7:**
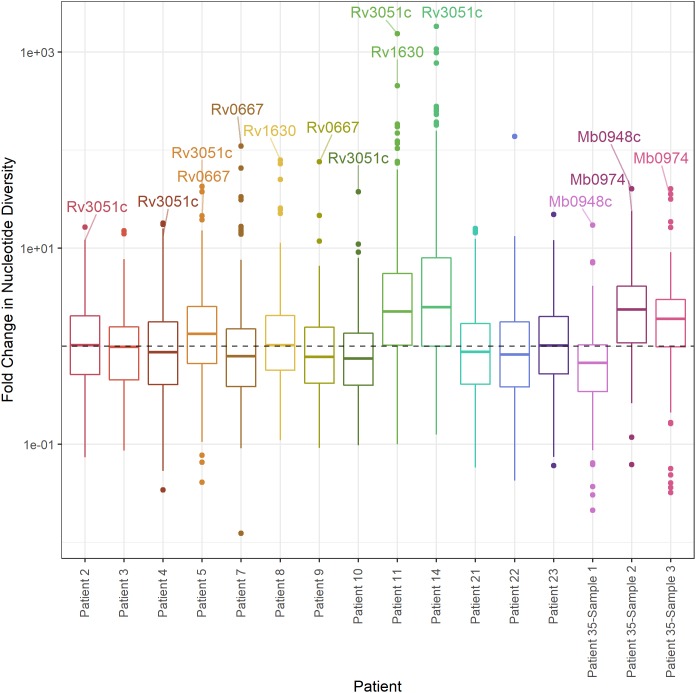
Distribution of fold change in nucleotide diversity per gene. Boxplots of fold change in nucleotide diversity per gene (π sputum/π culture). *Y*-axis log10 scaled. Outliers identified by *z*-score present in > 1 patient labeled (*p*-value < 0.05).

As an alternate method of identifying genes with significant differences in diversity, we calculated the fold-change in nucleotide diversity (π sputum/π culture) per gene in each patient and sample. Fold changes vary among intra- and inter-patient samples, and the fold changes from sputum to culture can span orders of magnitude (Figure 7). We calculated a *z*-score and *p*-value for the fold change per gene in each patient and identified three *M. tb* genes with significant fold change in > 1 patient; an additional 71 genes had an extreme fold change in a single patient (Figure 7, Table 1, and Supplementary Table S6).

Of these outliers, *nrdE* has the highest fold-change in four patients and *rpoB* (Rv0667) in two patients. *nrdE* is seen in the extremes of the fold-change distributions in a total of six patients, and *rpoB* in two. Although it is only found in the extremes of diversity in a single patient, *plcC* is the only gene with higher diversity in sputum in all 13 TB patients.

To assess genes with high diversity in sputum that have zero diversity in culture, which aren’t amenable to fold-change calculation, we examined differences rather than fold-changes. For genes with non-zero diversity in sputum and zero diversity in culture we calculated a *z*-score and *p*-value for each difference in nucleotide diversity (π) and identified 127 *M. tb* genes with significant differences. Fifteen of these were found across more than one patient (Table 1 and Supplementary Table S6).

As another method of identifying genes with major changes in diversity, we calculated *F*_ST_ per gene treating sputum and culture as two populations. We used Fisher’s exact test (with FDR correction) to assess the significance of *F*_ST_ per gene and found 63 *M. tb* genes to have significant differences in more than one patient; an additional 292 genes were outliers in a single patient (Table 1, Supplementary Table S6, and Supplementary Figure S6).

## Discussion

Although *M. tb* can be grown axenically in the lab, its natural niche is within human tissues. Here we compared patterns of genetic diversity of *M. tb* in expectorated sputum to bacteria grown *in vitro*, in order to gain insight on differences between evolutionary pressures encountered within the host and those imposed by *ex vivo* manipulation of bacterial populations. It’s important to understand bacterial adaptation to both settings, as the former is informative of host-pathogen interactions and the latter is vital in distinguishing signal from noise in bacterial sequencing data. We found that diversity of *M. tb* in sputum samples varies substantially within and among hosts, and that diversity of these populations is higher than it is for *M. tb* grown *in vitro*. Using outlier analyses, we further identified a group of genes that exhibit consistent shifts in diversity between culture and sputum. These are candidate loci mediating differential adaptation to the two environments.

We found overall diversity of *M. tb* populations to be higher in sputum samples than in culture (Figure 2, 4). At a gene by gene level, the most common pattern observed was for genes with measurable diversity in sputum to lose all diversity in culture (Figure 5). This pattern could arise from bacterial population bottlenecks that occur during processing of sputum samples for *in vitro* culture. Sputum and culture samples from Brown et al. (2015) were produced from different input volumes of initial suspension (1900 and 100 ul, respectively). We controlled for this potential bias in the processing of samples from patient 35, using equal volumes of initial suspension for direct DNA extraction and inoculation into culture media. The results from patient 35 mirror those of the Brown samples, with greater diversity of *M. tb* in sputum than in culture (Figure 2, 4). This suggests that the reduction in diversity observed in culture samples is not an artifact of sample processing.

An alternate explanation of the observed difference between sputum and culture samples is that apparent diversity of *M. tb* in sputum samples is inflated by DNA sequences from organisms other than *M. tb*, i.e., bacteria present in the upper respiratory tract. We applied stringent filters to remove off-target sequences (see section “Materials and Methods”), and our analyses of uninfected sputa (Supplementary Table S3) and culture samples spiked with TB-negative sputa (see section “Results”, Supplementary Figure S1) showed that the patterns of *M. tb* diversity observed in sputum samples did not arise from contamination of *M. tb* sequencing data.

Explanations of the relatively high diversity in sputum include relaxed purifying selection and/or diversifying selection that is specific to this environment, mutation rate variation, and bacterial sub-populations within hosts that have variable fitness *in vitro*. The degree of differentiation between *M. tb* populations in sputum and culture varies substantially among patients (Figure 2, 6): overall diversity of bacterial populations in the two environments is nearly identical for some patients (e.g., patient 4) and an order of magnitude different for others (e.g., patient 14). This suggests that the evolutionary pressures driving genome wide differences in diversity vary from patient to patient.

We found previously, using pooled culture-based samples, that overall diversity of within-host *M. tb* populations varies among patients and that patients with pre-terminal TB can harbor extremely diverse populations of bacteria (O’Neill et al., 2015). It’s possible that *M. tb* populations within hosts occasionally undergo massive expansions associated with relaxation of purifying selection, and that this becomes evident in comparisons with bacterial populations cultured under relatively uniform conditions. Pulmonary cavitation is one plausible condition under which such an expansion could occur: cavitation results in a shift from a hypoxic to an oxygen-rich environment and the interior of the cavity is relatively inaccessible to the immune system. Trauner et al. (2017) reported an observable shift in *M. tb* population structure following cavitation of a large granuloma, demonstrating that *M. tb* within-host population diversity reflects the ongoing evolution of disease in the host. Clinical metadata from the patients whose samples we analyzed here do not point to any obvious reasons for observed differences in *M. tb* sputum diversity [e.g., patient 4, with low diversity, has 3+ smear positivity and MDR TB and patient 14, with high diversity, has 1+ smear and MDR TB; (Brown et al., 2015)], but these data are limited.

Host immune responses impose a range of stresses on *M. tb* populations, including DNA damage (reviewed in Stallings and Glickman, 2010; Flentie et al., 2016). Host imposed mutagenic stressors are likely to vary over time and among patients, and high *M. tb* sputum diversity could reflect more mutagenic environments within certain hosts (and host states) versus the relatively uniform conditions of *in vitro* culture. Relatively high diversifying selection is an alternative explanation for high *M. tb* sputum diversity within a subset of TB patients. However, given that the pattern of elevated diversity is genome-wide (Supplementary Figure S2), this seems less likely than relaxed purifying selection and/or variation in within-host mutation rates.

Beyond its potential instructiveness about the varied adaptive milieu within hosts with TB, the uneven accumulation of *M. tb* genetic diversity across TB patients has implications for the reconstruction of TB transmission networks from bacterial genetic data. *M. tb* genetic distances have been used as evidence of epidemiological links among TB patients and method development is active in this area (e.g., Stimson et al., 2019). Our finding here and in prior published work that *M. tb* diversity varies dramatically within patients with TB implies that epidemiological links can be obscured in pathogen genetic data. In a recently published study comparing *M. tb* outbreak strains with endemically circulating strains, we found evidence suggesting that bacterial diversification is uneven, characterized by long periods of stasis and punctuated bursts (Doroshenko et al., 2018). This pattern could arise from occasional, exceptionally large bacterial population expansions and/or mutation rate variation within hosts.

Our finding of increased *M. tb* diversity in sputum relative to culture is consistent with results of other studies using capture based methods (Doyle et al., 2018; Nimmo et al., 2018). Votintseva et al. (2017), who used shotgun sequencing to compare *M. tb* in sputum and culture, did not identify a difference in overall diversity between these sample types. The shotgun and capture-based studies are not directly comparable, as shotgun sequencing is less sensitive and was applied to smear positive samples only. In addition, coverage was inadequate to allow diversity to be estimated for several of the samples in Votintseva et al. (2017).

Variant calling and quantification of diversity was also performed differently across studies. Nimmo et al. estimated the number of heterozygous sites, as did Votintsteva et al., but Votintseva et al. used a distinct variant calling method and restricted their analysis to a subset of 68,695 loci at which they had previously identified segregating polymorphisms in a large sample of clinical isolates. Culture-based studies of intra-host diversity suggest that most *M. tb* variants are segregating at rare frequencies (O’Neill et al., 2015; Trauner et al., 2017), which parallels findings at the between-host scale (Pepperell et al., 2013). Our findings here also suggest that within-host diversity is skewed to rare variation, and that this skew is more pronounced in sputum than in culture (Supplementary Figure S4). There is no *a priori* reason to expect that the same rare mutations will be encountered in individual clinical isolates, culture-based surveys of within-host diversity, and clinical samples. Based on these observations, we posit that restricting the estimation of *M. tb* diversity in sputum to loci at which variants were observed in clinical isolates is likely to result in an underestimate of the amount of bacterial variation present in sputum.

Results from several studies suggest that the *M. tb* population within hosts is structured into genetically distinct sub-populations (Liu et al., 2015; Lieberman et al., 2016; Martin et al., 2017; Trauner et al., 2017). Consistent with these prior studies, our results here demonstrate sample to sample variation in sputa collected from a single patient (Supplementary Table S2 and Figure 3).

Published data demonstrate that sputum from TB patients contains phenotypically distinct sub-populations of *M. tb* and that these phenotypes are not recovered during *in vitro* culture (Garton et al., 2008). *In vitro* culture of mixtures of genetically and phenotypically distinct *M. tb* has been shown to result in a loss of diversity (Martín et al., 2010; Hanekom et al., 2013; Metcalfe et al., 2017) and *M. tb* adaptation to laboratory conditions is a well described phenomenon (Domenech and Reed, 2009; Ioerger et al., 2010; Molina-Torres et al., 2010; Domenech et al., 2014; De Majumdar et al., 2019). Taken together, these findings show that the population of *M. tb* within hosts is genetically and phenotypically diverse, and that *in vitro* culture imposes distinct evolutionary pressures on *M. tb* that reshape the bacterial population. It follows that the full diversity of *M. tb* found in sputum is unlikely to survive the transition to growth *in vitro*; this offers a complementary/ alternative explanation of observed differences in *M. tb* genetic diversity between sputum and culture.

In order to gain insight on evolutionary pressures in sputum and culture, we performed outlier analyses of gene-wise patterns of variation (Figure 6, 7 and Table 1). We identified two major groups of outlier genes. The first group, typified by Rv2020c (encoding a conserved hypothetical protein), exhibited high diversity in both sputum and culture without significant differences between environments (Figure 6). Genes with a similar pattern include two predicted adenylate cyclases (Rv1318c and 1319c), molybdenum cofactor biosynthesis protein *moaA1* (Rv3109), transcriptional regulatory protein *embR* (Rv1267c), membrane-associated phospholipases C1 and C2 (*plcB*/Rv2350c and *plcA*/Rv2351c), Rv2081c (conserved transmembrane protein) and Rv2082 (conserved hypothetical). We previously found Rv2020c to be in the 99th percentile of diversity in a sample of 201 globally extant strains of *M. tb* (O’Neill et al., 2015). Several other genes in this group exhibited similarly high diversity in our previous study: *plcA*/Rv2351c, Rv1319c, Rv2o81c, and Rv2082 were also in the 99th percentile of gene-wise diversity, whereas *plcB*/Rv2350c was in the 81st and *moaA1*/Rv3109 in the 87th percentile of gene-wise diversity in the global sample. With the exception of *moaA1*/Rv3109, for which data are conflicting, none of the genes in this grouping is essential for growth *in vitro* (Sassetti et al., 2003; Griffin et al., 2011; DeJesus et al., 2017). Deletions affecting *plcA*/Rv2351c have been identified in clinical *M. tb* isolates, suggesting its function is dispensable in certain settings and/or genetic backgrounds (Tsolaki et al., 2004). Collectively, these results suggest the genes are under relaxed purifying selection or diversifying selection.

Interestingly, for three of the six genes in this group (*plcA*/Rv2351c, *plcB*/Rv2350c, Rv2020c), growth *in vitro* is actually enhanced when the gene is disrupted by transposon insertion (DeJesus et al., 2017); this is also true of *plcC*/Rv2349c, which was not an outlier but exhibited consistent differences between sputum and culture. Gene expression studies suggest that *M. tb* in sputum are in a slowly replicating or non-replicating state relative to *M. tb* in culture (Garton et al., 2008; Honeyborne et al., 2016; Sharma et al., 2017). Non-replicating persistence is likely adaptive, as *M. tb* in this physiological state is able to survive a wide range of stressors and becomes progressively enriched in the sputa of TB patients (Betts et al., 2002; Voskuil et al., 2004; Honeyborne et al., 2016). A recent, detailed investigation of persistent *M. tb* in sputum identified several distinct sub-populations of bacteria, suggesting that selection for this trait maintains diversity in natural populations of *M. tb* (Jain et al., 2016). We found previously that a subset of positively selected loci in *M. tb* are characterized by high diversity and numerous rare mutations; we referred to these loci as “sloppy targets” (Mortimer et al., 2018). Here we propose that Rv2020c, Rv1318c, Rv1319c, Rv2081c, Rv2082, *moaA1*/Rv31009, *embR*/Rv1267c, *plcA*/Rv2351c, and *plcB*/Rv2350c are sloppy targets. Of note, similar to *plcA*/Rv2351c, the canonical sloppy target *pncA* is deleted in commonly circulating sub-lineages of *M. tb* (Nguyen et al., 2003, 2004). The nine putative sloppy targets identified in this study were all FST outliers in multiple patients, indicating that while these genes are similarly diverse in sputum and culture, variants within them differ between environments (Table 1). This is consistent with positive selection in at least one of these environments as an explanation of high diversity, as opposed to global relaxation of purifying selection. Selection for persistence is a possible example of differential selective pressure in sputum and culture as this trait is unlikely to be adaptive during growth in antibiotic free media.

We identified a second group of genes, typified by ribonucleoside diphosphate reductase *nrdE* (Rv3051c), characterized by marked changes in diversity between sputum and culture. Genes in this group, which we will hereafter refer to as “shifting targets”, include RNA polymerases *rpoB* (Rv0667) and *rpoC* (Rv0668), elongation factor *fusA1* (Rv0684), ribosomal protein *rpsA* (Rv1630), iron sulfur binding reductase Rv0338c, respiratory nitrate reductase *narI* (Rv1164), and maltosyltransferase *glgE* (Rv1327c). Genes in this grouping are annotated as either intermediary metabolism (*n* = 3) or information pathways (*n* = 5); all but one (*narI*/Rv1164) is essential for *in vitro* growth. *RpoB* and *rpoC* are known to mediate resistance to rifamycins, which are first line TB treatments; as expected, signatures of positive selection have been identified previously at these loci (Mortimer et al., 2018; Wilson and Consortium, 2019). TB treatment details were not provided for the samples included in Brown et al. (2015), but at least some of the patients included in the study and analyzed here had been treated previously. We clearly expect selection pressures on drug resistance loci to shift between sputum and culture in antibiotic free media, and thus the identification of *rpoB* and *rpoC* provides support for the use of our outlier method to identify genes under differential selection pressures *in vivo* and *in vitro*. As with the putative sloppy targets, the eight genes listed above were *F*_ST_ outliers across multiple patients (Table 1), further supporting the idea that they are under distinct selection pressures in the two environments. Of note, seven of eight genes in this group (*glgE*/Rv1327c is the exception) appear to be expressed differently in sputum versus culture (Garton et al., 2008; Garcia et al., 2016; Honeyborne et al., 2016; Sharma et al., 2017). As described above, broad patterns of gene expression suggest that the shift of *M. tb* from sputum to culture involves an increase in metabolic activity and replication. We hypothesize that shifting targets are under relatively strong purifying selection *in vitro*, as bacteria compete in an environment in which it is no longer advantageous to suspend growth. This transition to relatively strong purifying selection is expected to result in a decrease in diversity during culture, as observed here.

In this analysis of *M. tb* and *M. bovis* genomic data recovered directly from sputum and from cultured samples, we identified intra- and inter-patient variability, as well as an effect of sample type on bacterial genetic diversity. We hypothesize that this variability reflects differences in the milieu within hosts, the nature of host pathogen interactions, and the distinct evolutionary pressures experienced by these bacteria in natural and laboratory environments.

## Data Availability

The datasets generated for this study can be found in NCBI, PRJNA532927.

## Author Contributions

CP conceived the study. JD performed the sample preparation and processing. AS, JD, and CP designed the analyses, analyzed the data, and drafted the manuscript.

## Conflict of Interest Statement

The authors declare that the research was conducted in the absence of any commercial or financial relationships that could be construed as a potential conflict of interest.

## Footnotes

1 https://github.com/pepperell-lab/RGAPepPipe

2 https://github.com/FelixKrueger/TrimGalore

3 https://broadinstitute.github.io/picard/
