# Supplementary material for: Effects of Host, Sample, and in vitro Culture on Genomic Diversity of Pathogenic Mycobacteria
Source: Front Genet. 2019 Jun 4;10:477. doi: 10.3389/fgene.2019.00477 (PMC6558051; doi:10.3389/fgene.2019.00477)
Supplement: Supplementary file 7 [file Data_Sheet_1.docx]

**TABLE OF CONTENTS: SUPPLEMENTAL MATERIAL**

1. Table S1: Map of regions removed from *M. tuberculosis* and *M. bovis* alignments
2. Table S2: Variation at lineage defining loci
3. Table S3: Coverage statistics and lineage typing for all samples
4. Table S4: ANOVA analysis of coverage versus smear status
5. Table S5: High diversity genomic windows shared among samples
6. Table S6: Outlier genes
7. Figure S1: Sliding window Pi
8. Figure S2: Sliding window Theta
9. Figure S3: Sliding window Tajima’s D
10. Figure S4: Sliding window diversity of Patient 14 in sputum, culture and culture spiked with negative sputum
11. Figure S5: Sliding window fold change
12. Table S5: Genes with extreme absolute differences between sputum and culture
13. Table S6: F_ST_ outlier frequency
14. Figure S6: Histogram of F_ST_ outliers in *M. tuberculosis* samples.


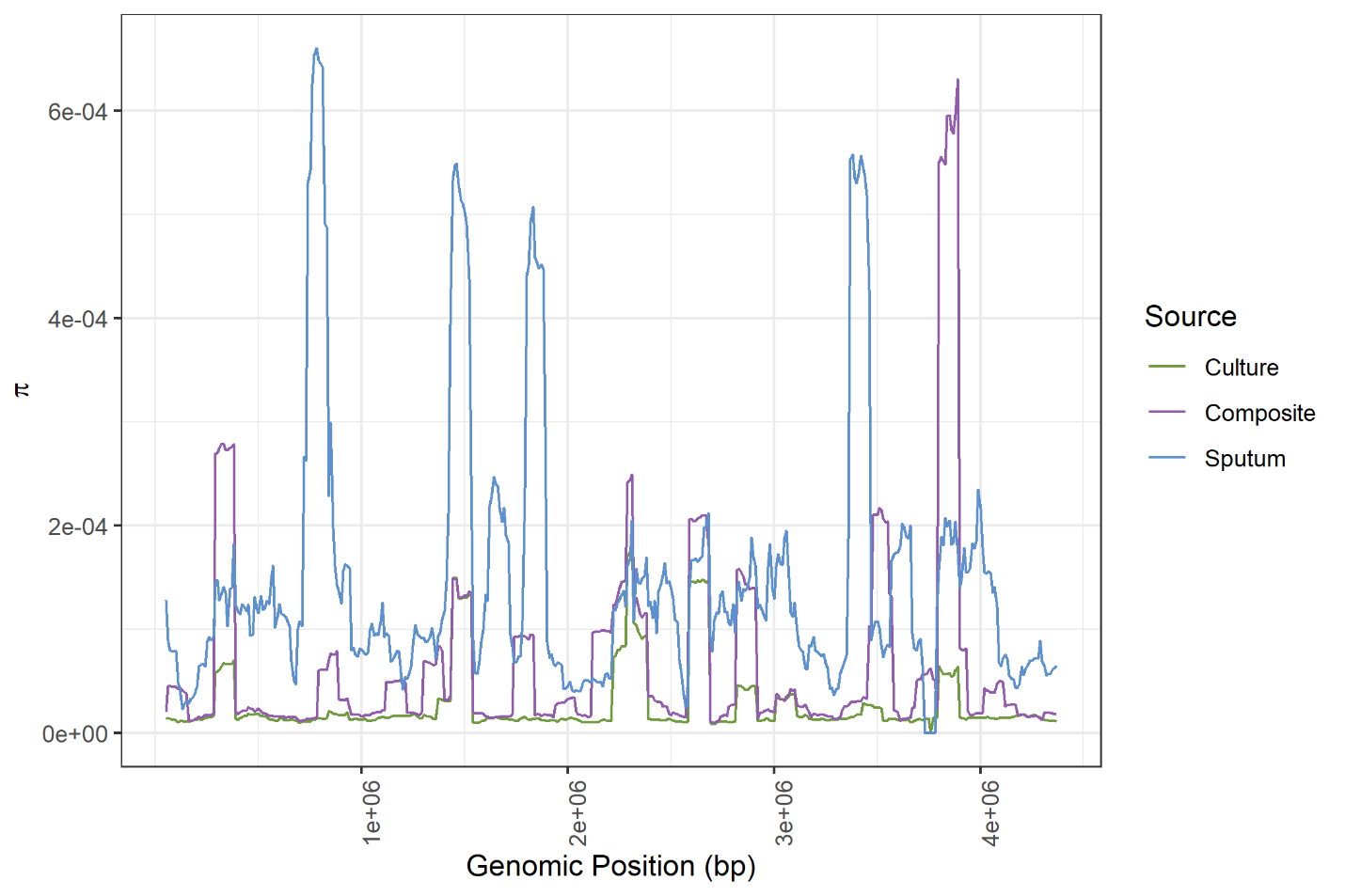


**Figure S1. Sliding window diversity of Patient 14 in sputum, culture and culture spiked with negative sputum**. Nucleotide diversity (π, y-axis) calculated in sliding windows (10 kb steps, 100 kb windows) across the genome (x-axis) for the culture, composite, and sputum samples from Patient 14. Composite sample contains sequences from Patient 14’s culture sample and ~3,000 sequences from TB negative sputum that passed metagenomic filtering. Culture, composite and sputum pictured in green, purple and blue, respectively. Background contamination does increase nucleotide diversity across the genome, but the patterns produced do not mirror those seen in the sputum sample.


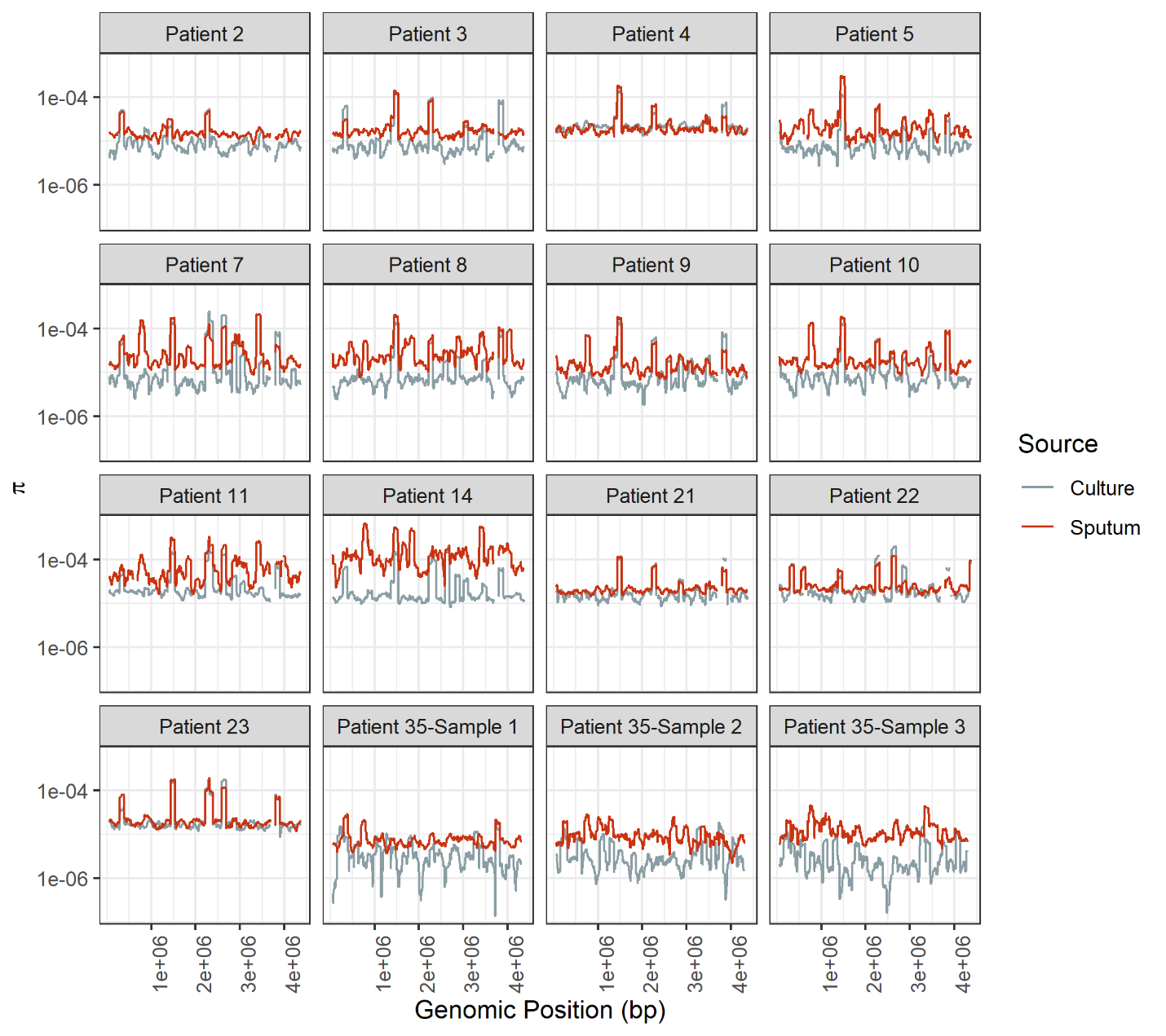


**Figure S2. Patterns of nucleotide diversity in composite patient sample.** Nucleotide diversity (π_,_ y-axis log10 transformed) calculated in sliding windows (10 kb steps, 100 kb windows) across the genome (x-axis) for sputum and culture samples from each patient.


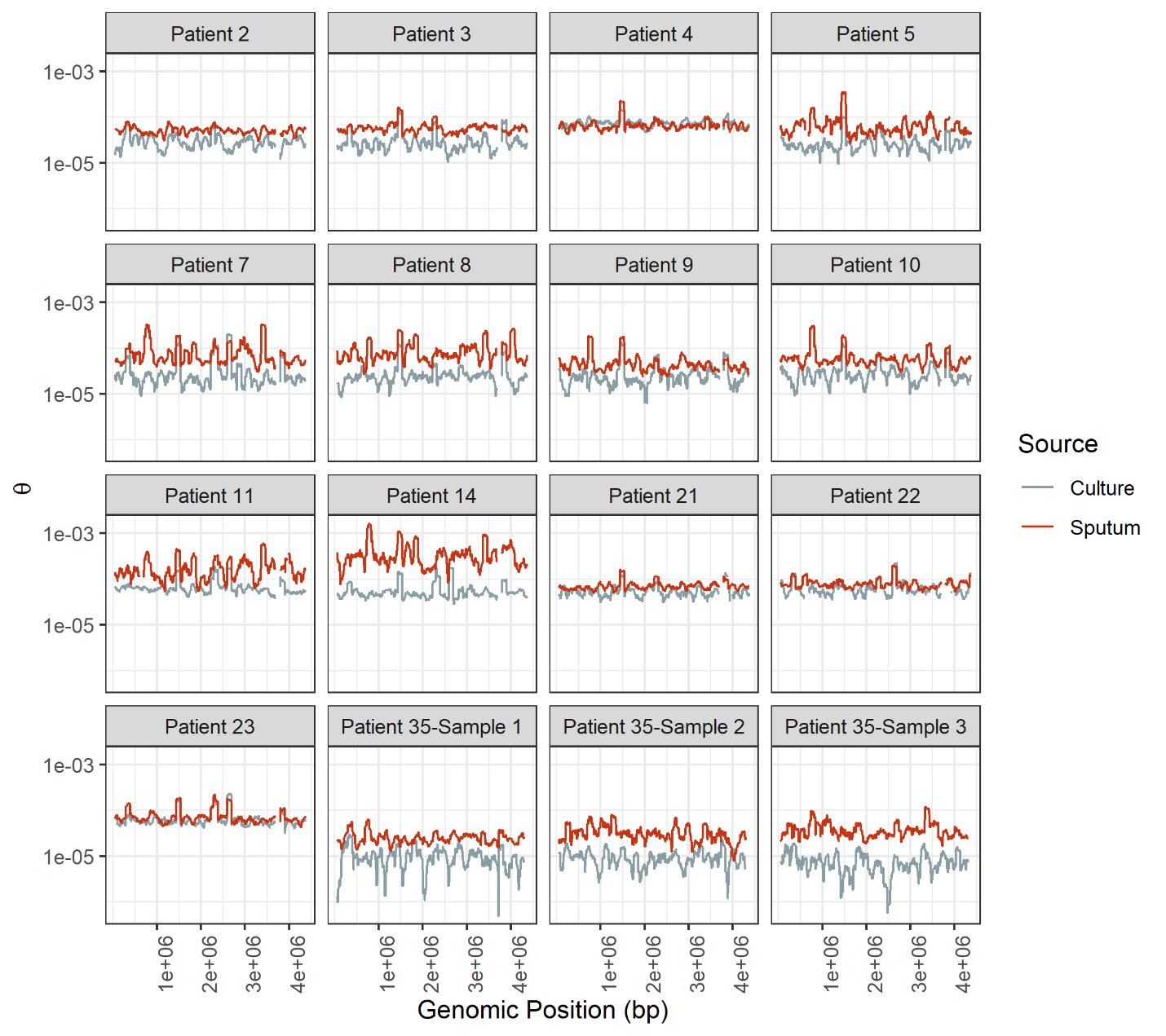


**Figure S3. Patterns of Watterson’s theta in sputum and culture.** Watterson’s theta (θ_W,_ y-axis log10 transformed) calculated in sliding windows (10 kb steps, 100 Kb windows) across the genome (x-axis, in bp) for sputum and culture samples from each patient.


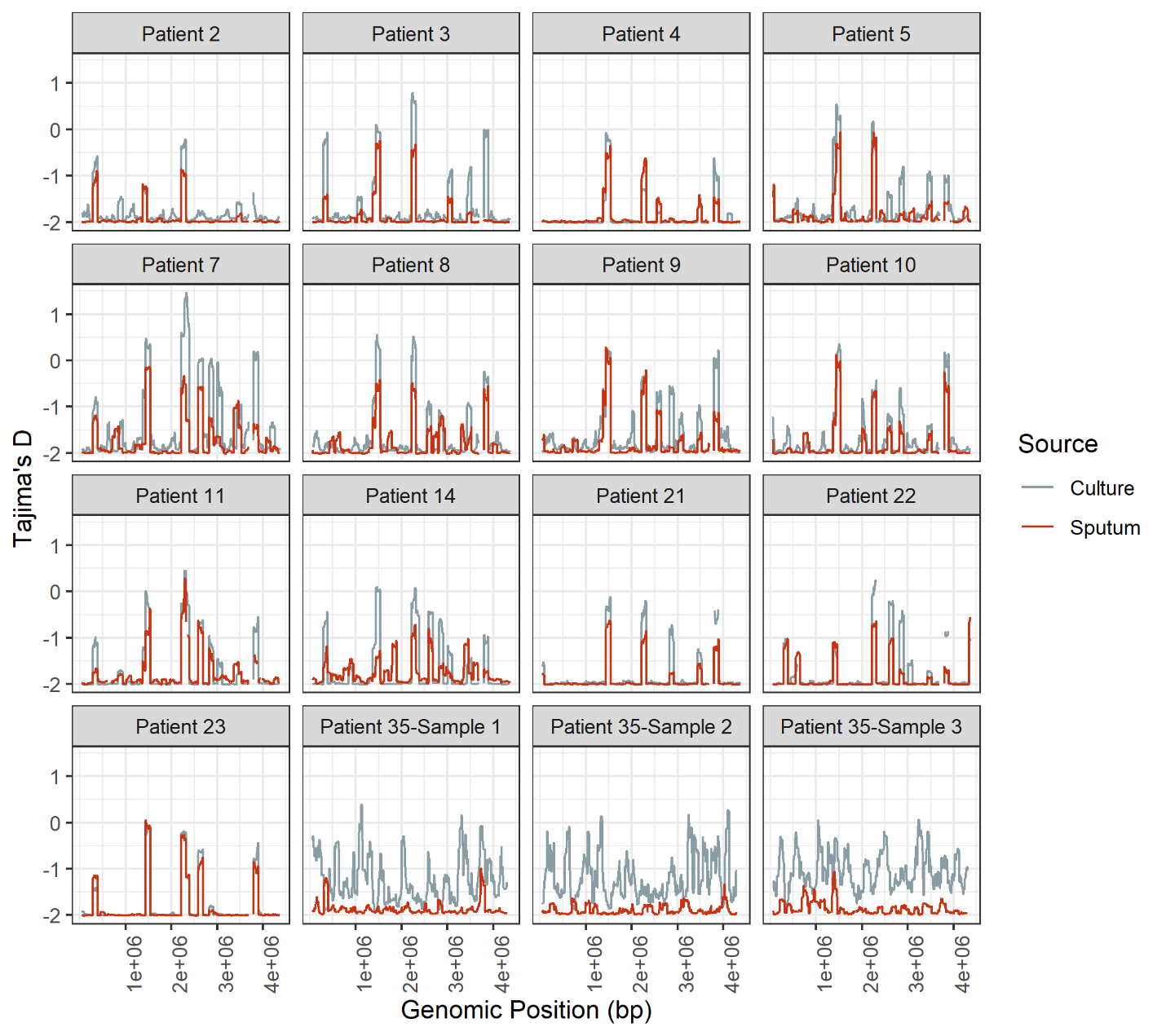


**Figure S4. Patterns of Tajima’s D in sputum and culture.** Tajima’s D (y-axis) calculated in sliding windows (10 kb steps, 100 Kb windows) across the genome (x-axis, in bp) for sputum and culture samples from each patient.

**
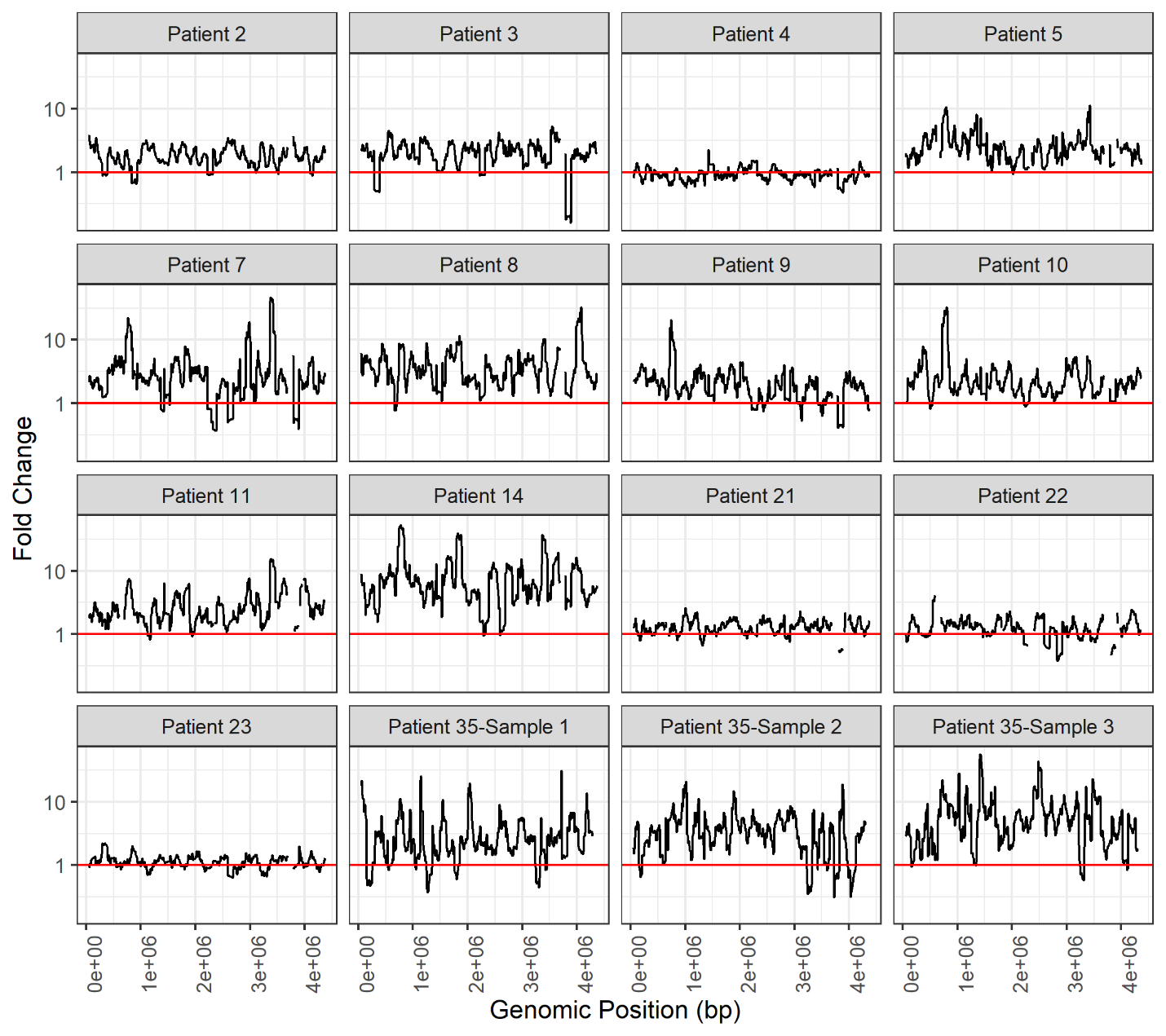
**

**Figure S5. Patterns of fold change in nucleotide diversity across the genome.** Fold change in nucleotide diversity **(**y-axis log10 transformed) from sputum to culture across the genome (x-axis, in bp) for each patient. Fold change calculated from sliding-window analysis of nucleotide diversity as π per window in sputum/π per window in culture for each patient.

**
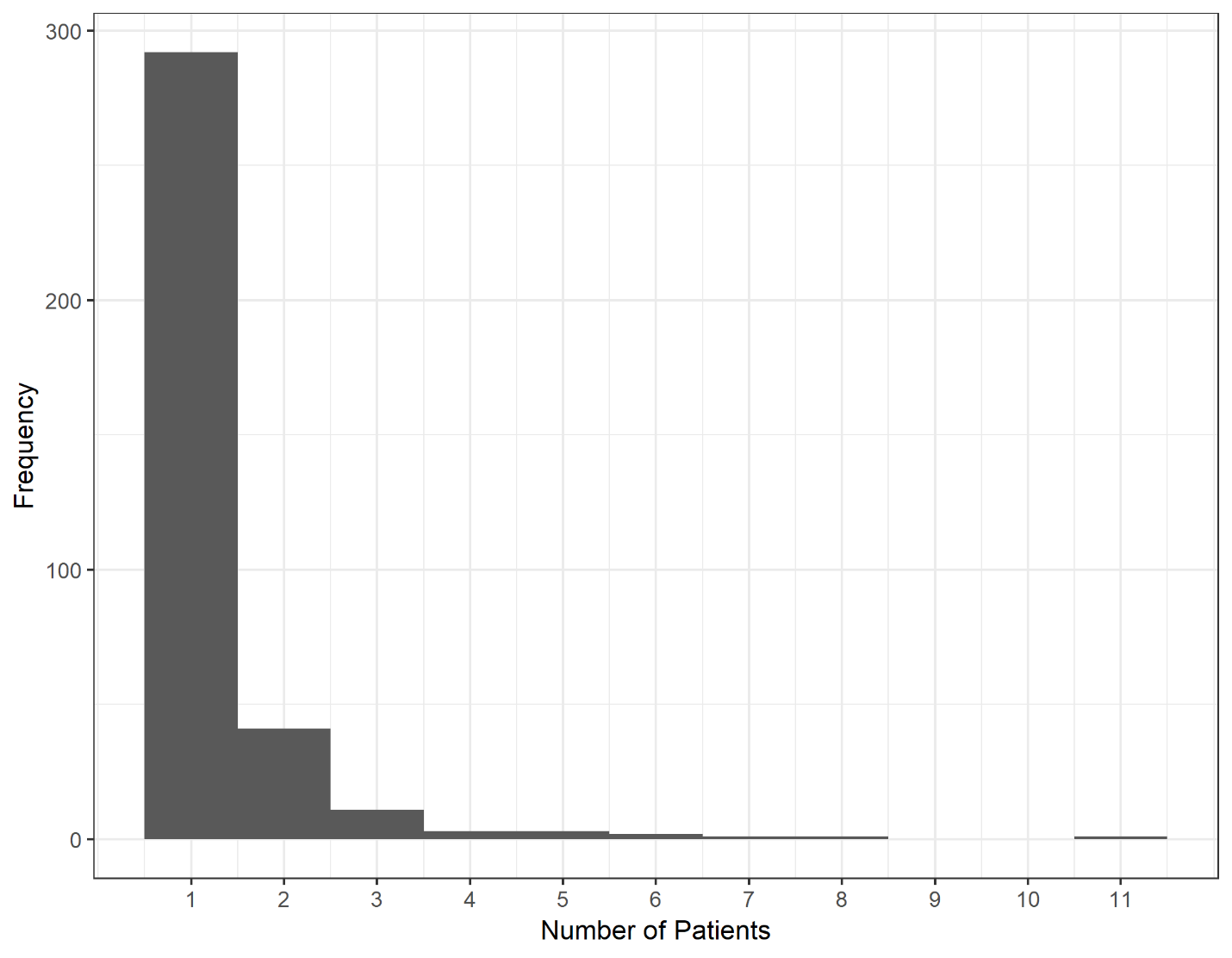
**

**Figure S6. Histogram of F_ST_ outliers in *M. tuberculosis* samples.** Frequency (y-axis) of F_ST_ outliers versus number of patients in which an outlier is present (x-axis). F_ST_ outliers identified using Fisher’s exact test (adjusted p-value < 0.01).
